# Prostaglandins Regulate Urinary Purines by Modulating Soluble Nucleotidase Release in the Bladder Lumen

**DOI:** 10.3390/ijms26168023

**Published:** 2025-08-19

**Authors:** Mahsa Borhani Peikani, Alejandro Gutierrez Cruz, Zoe S. Buckley, Violeta N. Mutafova-Yambolieva

**Affiliations:** Department of Physiology and Cell Biology, School of Medicine, University of Nevada Reno, Reno, NV 89557, USA

**Keywords:** nucleotidases, prostaglandins, ATP, bladder, urothelium, prostaglandin D_2_, prostaglandin I_2_, prostaglandin E_2_

## Abstract

Distention of the urinary bladder wall during filling stretches the urothelium and induces the release of chemical mediators, including adenosine 5′-triphosphate (ATP) and prostaglandins (PGs), that transmit signals between cells within the bladder wall. The urothelium also releases soluble nucleotidases (s-NTDs) that control the availability of ATP and its metabolites at receptor sites in umbrella cells and cells deeper in the bladder wall, as well as in the urine. This study investigated whether PGs regulate the intravesical breakdown of ATP by s-NTDs. Using a murine decentralized mucosa-only bladder model and an HPLC technology with fluorescence detection, we evaluated the decrease in ATP and increase in ADP, AMP, and adenosine (ADO) in intraluminal solutions (ILS) collected at the end of physiological bladder filling. PGD_2_, PGE_2_, and PGI_2_, but not PGF_2α_, inhibited the conversion of AMP (produced from ATP) to ADO, likely due to a suppressed intravesical release of s-AMPases. The effects of exogenous PGD_2_, PGE_2_, and PGI_2_ were mediated by DP1/DP2, EP2, and IP prostanoid receptors, respectively. Activation of either DP1 or DP2 receptors by endogenous PGD_2_ also led to AMP increase and ADO decrease in ILS-containing ATP substrate. Finally, PGs produced by either COX-1 or COX-2 inhibited the hydrolysis of AMP to ADO. Together, these observations suggest that (1) endogenous PGs (chiefly PGD_2_, and to lesser degree PGE_2_ and PGI_2_) allow release of s-NTDs like s-ATPases and s-ADPases but impede the formation of ADO from intravesical ATP by inhibiting the release of s-NTDs/s-AMPases; (2) it is possible that high concentrations of PGD_2_, PGE_2_ and PGI_2_, as anticipated in inflammation or bladder pain syndrome, delay the ADO production and prolong the action of excitatory purine mediators; and (3) either COX-1 and COX-2 are constitutively expressed in the mouse bladder mucosa or COX-2 is induced by distention of the urothelium during bladder filling.

## 1. Introduction

During filling of the bladder with urine, adenosine 5′-triphosphate (ATP) is released from the bladder mucosa in both lamina propria (LP) and bladder lumen [1,2], along with soluble nucleotidases (s-NTDs) that sequentially hydrolyze ATP to adenosine 5′-diphosphate (ADP), adenosine 5′-monophosphate (AMP), and adenosine (ADO) [3,4]. ATP that is released from the bladder mucosa is considered to be a key factor in the urotheliogenic control of bladder excitability [5,6]. In particular, it is assumed that the ATP that is released into the LP activates P2X2/P2X3 receptors on sensory neurons at the LP/urothelium interface to convey signals resulting from bladder wall distention to the CNS and trigger voiding [7,8]. The ATP that is released into LP also affects the functions of other cell types within the bladder wall, including interstitial cells [9] and detrusor smooth muscle cells [10]. Finally, ATP in the LP can also regulate bladder excitability by inducing or modulating the release of other mediators in the bladder mucosa, such as acetylcholine [11], nitric oxide [12], and prostaglandins (PGs) [13,14]. The physiological role of the ATP that is released from the bladder mucosa into the bladder lumen is less clear. It is proposed that intraluminal ATP and its primary product ADO are involved in the regulation of the uroepithelial surface area as the bladder stretches during filling [15,16]. Other reports suggest that intraluminal ATP might activate bladder afferent neurons in the urothelium and LP [8,17]. Most prominently, urinary ATP has been suggested as a biomarker of overactive or underactive bladder (OAB and UAB) dysfunction [18,19]. For instance, ATP is increased in the urine of patients with interstitial cystitis, bladder pain syndrome, and OAB [20,21], whereas decreased ATP is found in the urine of patients with refractory detrusor overactivity and bacteriuria [22] and patients with undefined UAB [23]. The urinary levels of ATP are a result of released minus degraded ATP. Recently discovered soluble nucleotidases (s-NTDs) participate in intravesical ATP degradation [4], contribute to the elimination of excess ATP, and regulate the effective concentrations of ATP, ADP, and ADO at the receptor sites on umbrella cells or cells deeper within the bladder wall. The urothelial release of s-NTDs is regulated by multiple mechanisms, including PIEZO channels, pannexin 1, sensory neuropeptides, P2X receptors, adenylyl cyclase activation, and adenosine [3,4,24,25]. Moreover, it appears that the release of s-NTDs on the luminal and abluminal sides of the urothelium is under differential control [4].

PGs are lipid mediators that are synthesized in the bladder wall through the oxidation of arachidonic acid by cyclooxygenases (COX) and conversion of an intermediary PGH_2_ to PGE_2_, PGF_2α_, PGI_2_, and PGD_2_ by prostanoid synthases. PGs, like ATP and s-NTDs, are found released in the bladder lumen during bladder wall distention [26] and particularly during injury or inflammation [27,28]. Therefore, it is highly likely that ATP, s-NTDs, and PGs are simultaneously present in the bladder lumen and may modulate each other’s functions. We recently demonstrated that PGE_2_, PGF_2α_, and PGD_2_ facilitate the hydrolysis of ATP by s-NTDs in the mouse bladder LP by activating several G-protein-coupled prostanoid receptors [29]. It is currently unknown whether PGs also regulate the intraluminal degradation of ATP by s-NTDs. The present study aimed to fill such gaps in our knowledge.

We evaluated the degradation of 1,*N*^6^-etheno ATP (eATP), a fluorescent analog of ATP, in intraluminal solutions (ILS) collected at the end of physiological filling of bladder preparations treated with either PGE_2_, PGF_2α_, PGI_2_, or PGD_2_. Then, we evaluated whether the effects of PGs that altered the eATP hydrolysis were mediated by specific PG prostanoid receptors. Finally, we examined whether endogenous PGs that are produced by either COX-1 or COX-2 also regulate the intravesical release of s-NTDs and the consequent degradation of eATP.

## 2. Results

### 2.1. Time Course of s-NTDs-Mediated eATP Degradation to eADP, eAMP, and eADO in the Bladder Lumen

**Figure 1:**
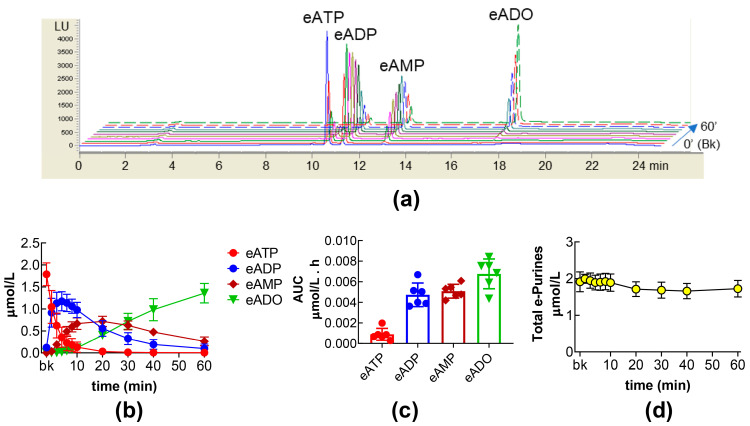
Time course of eATP degradation to eADP, eAMP, and eADO in ILS. Original HPLC chromatograms showing the hydrolysis of eATP and formation of eADP, eAMP, and eADO at 0′ (Bk, substrate), 10 s, 2′, 4′, 6′, 8′, 10′, 20′, 30′, 40′, and 60′ of contact of eATP with s-NTDs released in the bladder lumen during filling of the bladder with vehicle DMSO 0.2% (**a**). LU, luminescence units. Summarized results demonstrating the patterns of eATP decrease and eADP, eAMP, and eADO increase in ILS of bladders filled with vehicle, n = 6. Each purine is expressed as µmol/L detected in ILS at each time point of enzymatic reaction (**b**). Mean area under the curve (AUC) for time courses of eATP, eADP, eAMP, and eADO (**c**). Total e-purines (eATP + eADP + eAMP + eADO) in reaction solutions for the duration of enzymatic reactions (**d**).

The degradation of eATP by s-NTDs released in the bladder lumen during the filling of an ex vivo mouse bladder with a vehicle (i.e., DSMO 0.2%) is characterized by a rapid decrease of eATP, a rapid and transient increase of eADP, a gradual and more sustained increase of eAMP, and a gradual increase of eADO (Figure 1a,b). At 60 min of enzymatic reaction, the ILS collected at the end of bladder filling that was in contact with eATP contained approximately 20% eAMP and 80% eADO and almost no eATP or eADP (Figure 1b). Half of eATP was rapidly hydrolyzed within the first 10 s of contact with s-NTDs in the ILS, and eATP was completely degraded by the 10-min time point of the enzymatic reaction (Figure 1b). This was accompanied by a rapid and transient increase of eADP, reaching ~80% of its peak concentration in the first 10 s and its highest amounts at 4 min of reaction. This was followed by a gradual decrease of eADP to approximately 25%, 10%, and 2% of total purines at 30, 40, and 60 min of reaction (Figure 1b). In contrast to the time profiles for eATP and eADP, eAMP slowly accumulated to nearly 0.7 µmol/L (about 30% of total purines), then slowly declined to 20% of total purines at 60 min of reaction (Figure 1b). At 10 min of reaction, the eADO that was produced from eATP, eADP, and eAMP represented only 5% of the total purines in the reaction medium. This was followed by a gradual eADO increase, reaching 50% and 80% of total purines at 30 and 60 min of reaction, respectively. The AUC for the 60-min time course of eATP hydrolysis is shown in Figure 1c. It demonstrates that eATP was rapidly degraded by s-NTDs released in the bladder lumen during bladder filling and that eADO was the dominant eATP product. Notably, the concentration of total e-purines (i.e., eATP + eADP + eAMP + eADO) was close to the concentration of added eATP substrate throughout the 60 min period (Figure 1d), suggesting that eATP and its products eADP, eAMP, and eADO remained in the IL reaction solution and were not taken up by the cells present in ILS nor shifted to alternative metabolic pathways.

Due to the very rapid disappearance of eATP and the transient appearance of eADP, the drug-induced changes in eATP decrease and in eADP increase in IL reaction solutions could not be reliably detected. Instead, the slower time course of the eAMP and eADO increases permitted a more accurate evaluation of the sequential degradation of eATP to eAMP and eADO by released s-NTDs and the influences of pharmacological treatments.

### 2.2. Effects of PGE_2_, PGF_2a_, PGI_2_, and PGD_2_ on eATP Degradation by s-NTDs in the Bladder Lumen

**Figure 2:**
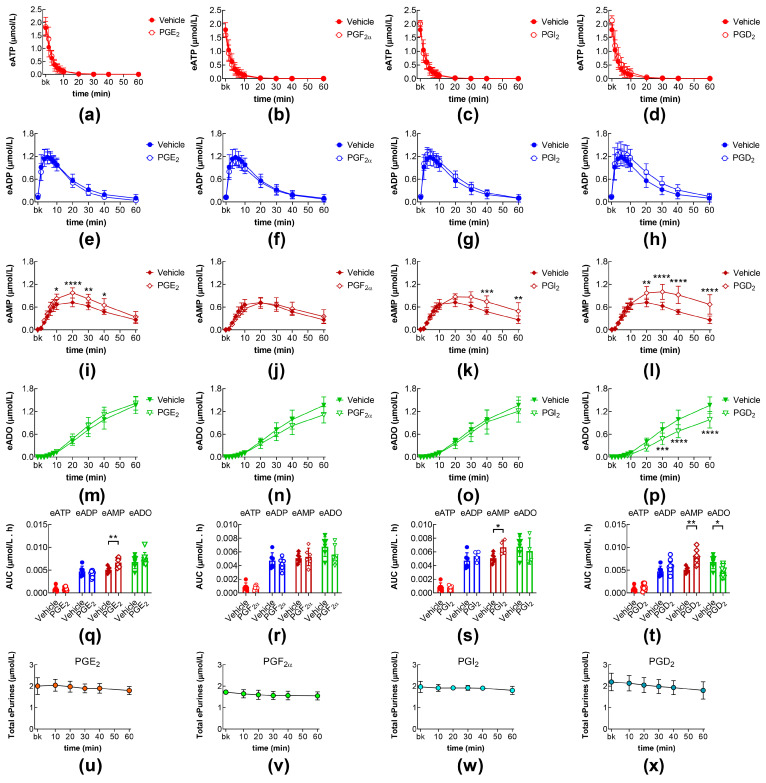
Effects of exogenous prostaglandins on the intravesical eATP hydrolysis. Time courses of the decrease of eATP (**a**–**d**) and the increase of eADP (**e**–**h**), eAMP (**i**–**l**), and eADO (**m**–**p**) in the presence of vehicle (n = 6) or PGE_2_ (n = 8) (**a**,**e**,**i**,**m**), PGF_2α_ (n = 6) (**b**,**f**,**j**,**n**), PGI_2_ (n = 4) (**c**,**g**,**k**,**o**), and PGD_2_ (n = 8) (**d**,**h**,**l**,**p**); n, number of bladder preparations. Each purine is expressed as µmol/L detected in ILS at each time point of enzymatic reaction. AUC for time courses of eATP, eADP, eAMP, and eADO (**q**–**t**). Asterisks denote significant differences from vehicle control. * *p* < 0.05, ** *p* < 0.01, *** *p* < 0.001, **** *p* < 0.0001. Sum of eATP, eADP, eAMP, and eADO (total e-purines) at each time point of enzymatic reactions in ILS of bladders treated with exogenous PGs (**u**–**x**).

First, we sought to determine whether high concentrations of exogenous PGs, as expected in bladder inflammation or injury of the bladder mucosa [27,30], affect the release of s-NTDs in the bladder lumen during filling and the consequent degradation of ATP. In these studies, we filled bladder preparations with a physiological Krebs (KBS) solution containing either a vehicle (DMSO at a final concentration of 0.2%) or PGE_2_, PGF_2α_, PGI_2_, or PGD_2_, each at a concentration of 10 µM. The most pronounced effects on eATP degradation were observed in ILS collected from bladders treated with PGD_2_, in which eAMP was significantly increased, whereas eADO was significantly decreased (Figure 2l,p,t). Similar, but weaker, alterations in eAMP concentrations were observed in the ILSs of bladder preparations treated with either PGE_2_ (Figure 2i,m,q) or PGI_2_ (Figure 2k,o,s). No significant changes in eATP degradation occurred in the ILSs of bladders filled with PGF_2α_ (Figure 2b,f,j,n,r). These results suggest that exogenous PGD_2_ and, to a lesser degree, PGE_2_ and PGI_2_ regulate the release of s-NTDs in the bladder lumen during bladder filling and the consequent multi-step intravesical hydrolysis of ATP. The three PGs primarily affected the hydrolysis of eAMP by soluble AMPases (s-AMPases) released in the ILS. With all PG treatments, the concentration of total e-purines was close to the concentration of added eATP substrate throughout the 60 min period (Figure 2u–x).

### 2.3. Effects of PGE2 in the Presence of EP1, EP2, EP3, and EP4 Receptor Antagonists

**Figure 3:**
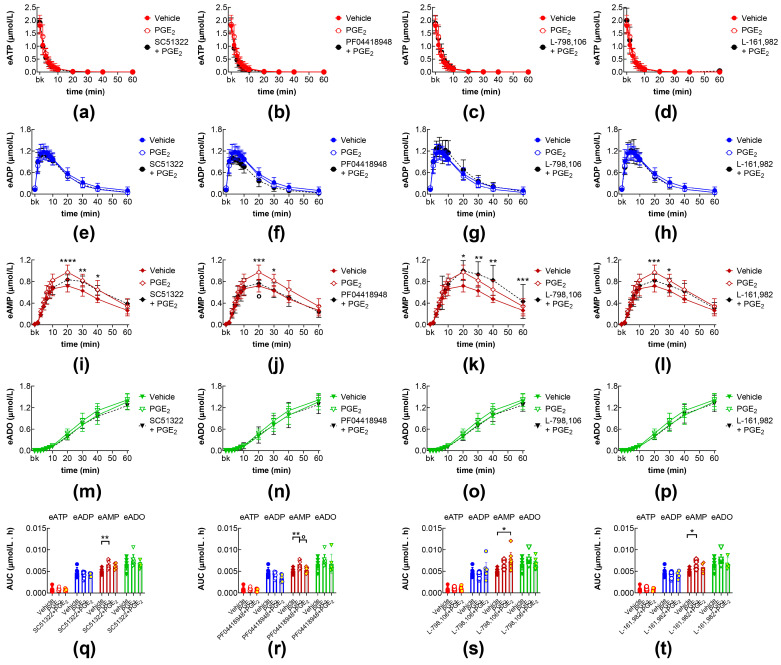
eATP hydrolysis by s-NTDs released in IL solutions of bladder preparations treated with exogenous PGE_2_ in the absence or presence of EP prostanoid receptor antagonists. Summarized results demonstrating time courses of eATP decrease (**a**–**d**) and the increase of eADP (**e**–**h**), eAMP (**i**–**l**), and eADO (**m**–**p**) by PGE_2_ (10 µM) in the presence of vehicle (n = 8) or of the EP1 antagonist SC51322 (1 µM, n = 6) (**a**,**e**,**i**,**m**), the EP2 receptor antagonist PF04418948 (1 µM, n = 6) (**b**,**f**,**j**,**n**), the EP3 receptor antagonist L-798,106 (0.25 µM, n = 10) (**c**,**g**,**k**,**o**), and the EP4 antagonist L-161,982 (1 µM, n = 4) (**d**,**h**,**l**,**p**); n, number of bladder preparations. AUC for time courses of eATP, eADP, eAMP, and eADO (**q**–**t**). Asterisks denote significant difference from vehicle control (n = 6). * *p* < 0.05, ** *p* < 0.01, *** *p* < 0.001, **** *p* < 0.0001. Open circle denotes significant differences in eATP degradation in PGE_2_ alone vs. EP receptor antagonist + PGE_2_. ^o^ *p* < 0.05. Two-way ANOVA with Tukey’s multiple comparisons test.

The effects of exogenous PGE_2_ on the eAMP and eADO increase were not affected by the pretreatment of the bladder lumen with the EP1 receptor antagonist SC51322 [31] (Figure 3a,e,i,m,q), the EP3 receptor antagonist L-798,106 [32] (Figure 3c,g,k,o,s), or the EP4 receptor antagonist L-161,982 [33] (Figure 3d,h,l,p,t). However, the increasing effect of exogenous PGE_2_ on eAMP produced from eATP hydrolysis was eliminated in the presence of the EP2 receptor antagonist PF04418948 [34] (Figure 3j,r).

### 2.4. Effects of PGI_2_ and PGD_2_ in the Presence of IP, DP1, and DP2 Receptor Antagonists

**Figure 4:**
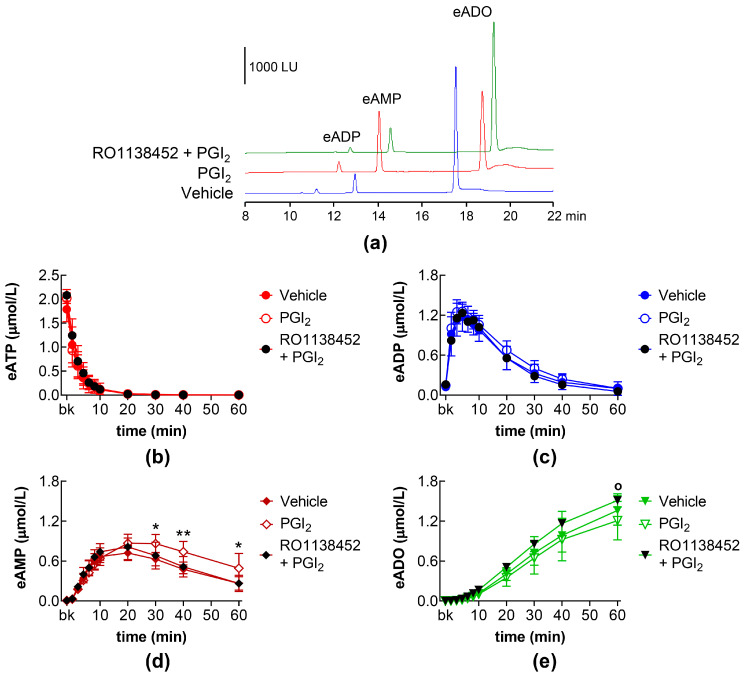
eATP hydrolysis by s-NTDs released in IL solutions of bladder preparations treated with PGI_2_ in the absence or presence of an IP prostanoid receptor antagonist. Original chromatograms showing the eATP degradation after 60 min of contact with IL solutions (**a**). LU, luminescence units. Summarized results demonstrating time courses of eATP decrease (**b**) and the increase of eADP (**c**), eAMP (**d**), and eADO (**e**) in ILS of bladders treated with vehicle (n = 6), PGI_2_ (n = 4) or RO1138452 + PGI_2_ (n = 4); n, number of bladder preparations. Asterisks denote significant differences vs. vehicle controls. * *p* < 0.05, ** *p* < 0.01. Open circle denotes significant differences in eATP degradation in PGI_2_ alone vs. RO1138452 + PGI_2_. ^o^ *p* < 0.05. Two-way ANOVA with Tukey’s multiple comparisons test.

The effects of exogenous PGI_2_ on the eAMP increase were abolished by pretreatment of the bladder lumen with the IP receptor antagonist RO1138452 (Figure 4d). Although the eADO production did not appear to be significantly affected by PGI_2_ treatment, treatments with RO1138452 + PGI_2_ did increase the formation of eADO, so that, at 60 min, eADO was significantly higher in the presence of RO1138452 + PGI2 than of PGI_2_ alone (Figure 4e).

**Figure 5:**
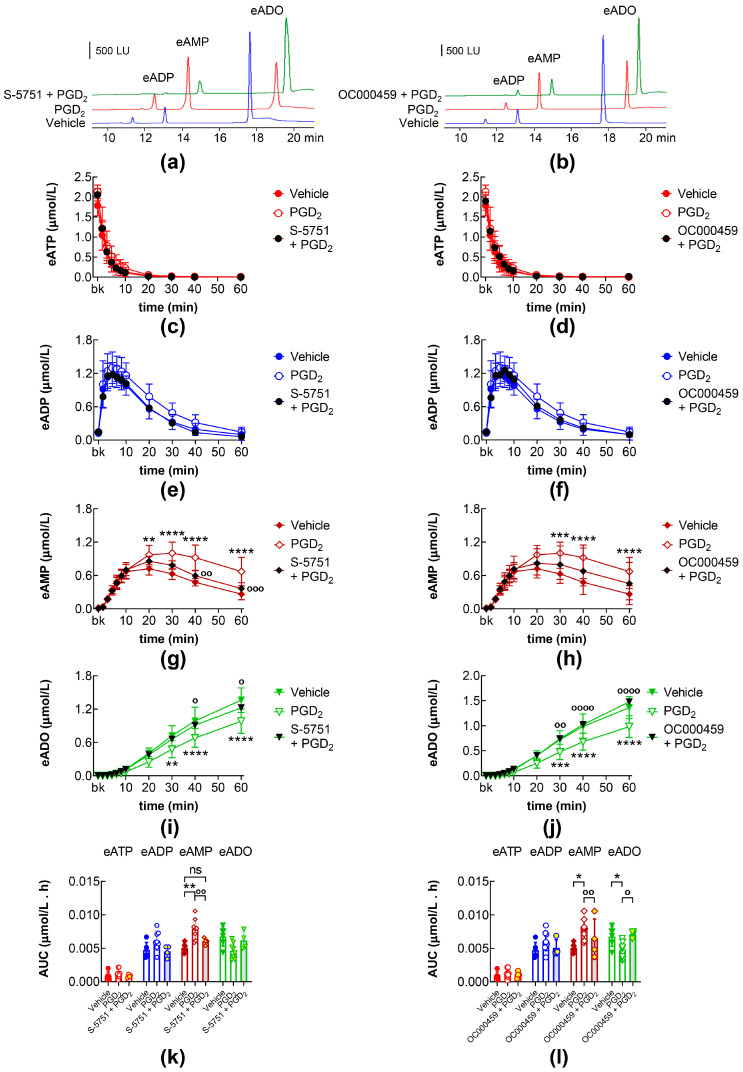
eATP hydrolysis by s-NTDs released in IL solutions of bladder preparations treated with PGD_2_ in the absence or presence of DP prostanoid receptor antagonists. Original chromatograms showing the eATP degradation after 60 min of contact with the ILS of bladders treated with PGD_2_ in the absence and presence of the DP1 receptor antagonist S-5751 (**a**) or of the DP2 receptor antagonist OC000459 (**b**). LU, luminescence units. Summarized results demonstrating time courses of eATP decrease (**c**,**d**) and the increase of eADP (**e**,**f**), eAMP (**g**,**h**), and eADO (**i**,**j**) in ILS of bladders treated with vehicle (n = 6), PGD_2_ (n = 8), S-5751 + PGD_2_ (n = 4) or OC000459 + PGD_2_ (n = 4); n, number of bladder preparations. AUC for time courses of eATP, eADP, eAMP, and eADO (**k**,**l**). Asterisks denote significant differences vs. vehicle controls. * *p* < 0.05, ** *p* < 0.01, *** *p* < 0.001, **** *p* < 0.0001, ns means not significant. Open circles denote significant differences in eATP degradation in PGD_2_ alone vs. DP receptor antagonist + PGD_2_. ^o^ *p* < 0.05, ^oo^ *p* < 0.01, ^ooo^ *p* < 0.001, ^oooo^ *p* < 0.0001. Two-way ANOVA with Tukey’s multiple comparisons test.

The eAMP increase and eADO decrease caused by PGD_2_ were inhibited by the DP1 receptor antagonist S-5751 (Figure 5a,g,i,k) and the DP2 receptor antagonist OC000459 (Figure 5b,h,j,l).

### 2.5. Effects of DP1 and DP2 Receptor Antagonists on Intraluminal eATP Degradation by s-NTDs

**Figure 6:**
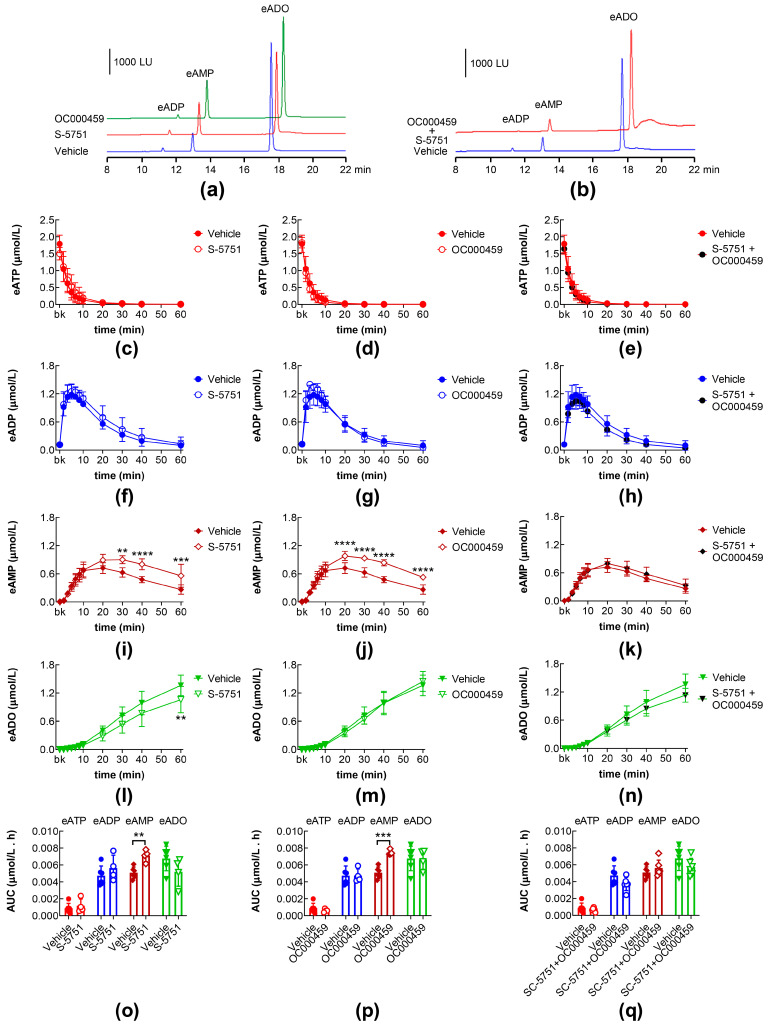
Effects of DP prostanoid receptor antagonists on the intravesical eATP hydrolysis. Original HPLC chromatograms showing the hydrolysis of eATP and formation of eADP, eAMP, and eADO after 60 min of contact of eATP with s-NTDs released in ILS of preparations treated either with vehicle (i.e., DMSO 0.2%) or with the DP1 receptor antagonist S-5751 and the DP2 receptor antagonist OC000459 (**a**) or S-5751 + OC000459 (**b**). LU, luminescence units. Summarized results demonstrating time courses of the eATP decrease (**c**–**e**) and the increase of eADP (**f**–**h**), eAMP (**i**–**k**), and eADO (**l**–**n**) in the presence of vehicle (n = 6) and S-5751 (n = 4) (**c**,**f**,**i**,**l**), OC000459 (n = 4) (**d**,**g**,**j**,**m**) or S-5751 + OC000459 (n = 4) (**e**,**h**,**k**,**n**); n, number of bladder preparations. Each purine is expressed as a percentage of the total amount of purines detected in ILS at each time point of enzymatic reaction. AUC for time courses of eATP, eADP, eAMP, and eADO (**o**–**q**). Asterisks denote significant differences from vehicle control ** *p* < 0.01, *** *p* < 0.001, **** *p* < 0.0001.

We next asked whether the endogenous ligand of the DP1 and DP2 receptors (i.e., endogenous PGD_2_) also regulates the release of s-NTDs. In the ILSs of bladders treated with the DP1 receptor antagonist S-5751 [35] alone, eAMP was increased and eADO was decreased at 30–60 min of reaction, in comparison with the vehicle controls (Figure 6a,i,l). In the ILSs of bladders treated with the DP2 receptor antagonist OC000459 [36] alone, the eAMP levels were increased (Figure 6a,j,p), but the eADO levels were unchanged (Figure 6a,m,p). In the ILSs of bladders treated simultaneously with S-5751 and OC000459, the eAMP and eADO production tended to return to control levels (Figure 6b,k,n,q). Therefore, the release of s-NTDs in the bladder lumen during filling appears to be regulated by endogenous PGD_2_.

### 2.6. Effects of COX-1 and COX-2 Inhibition on Intraluminal eATP Degradation by s-NTDs

**Figure 7:**
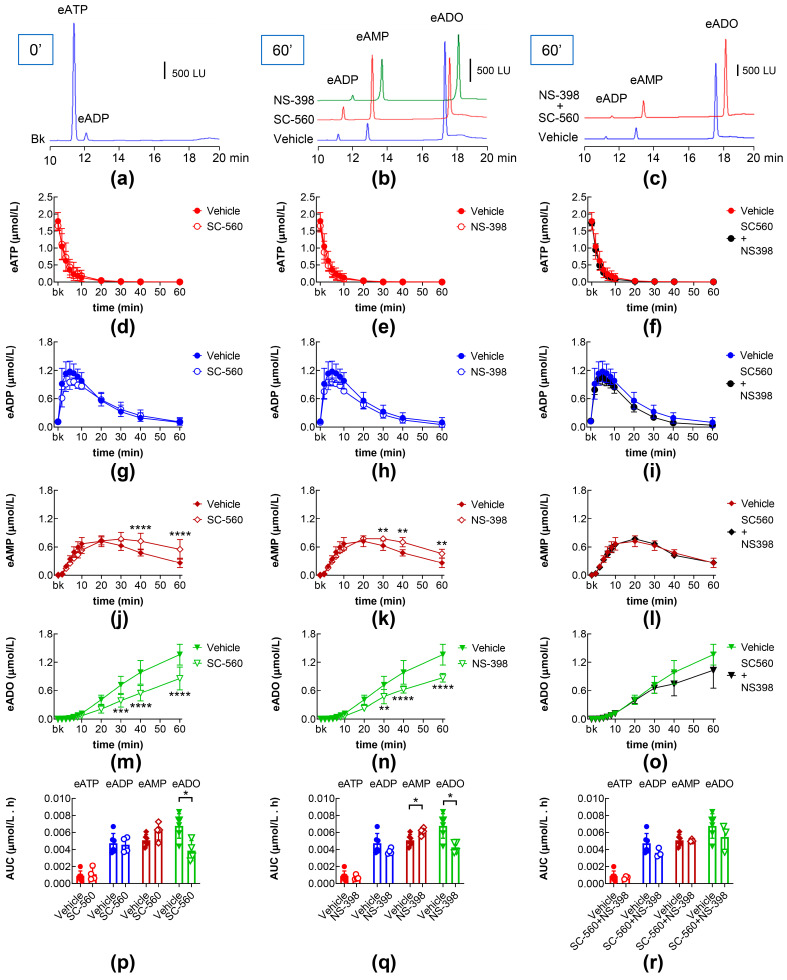
Effects of COX inhibitors on the intravesical eATP hydrolysis by s-NTDs. An original HPLC chromatogram of eATP substrate in the absence of enzymes (beaker, Bk) (**a**). Original chromatograms showing the hydrolysis of eATP and formation of eADP, eAMP, and eADO after 60 min of contact of eATP with ILS of bladders treated with vehicle, SC-560, NS-398 (**b**), or SC-560 + NS-398 (**c**). LU, luminescence units. Summarized results demonstrating time courses of the eATP decrease (**d**–**f**) and the increase in eADP (**g**–**i**), eAMP (**j**–**l**), and eADO (**m**–**o**) in the presence of vehicle (n = 6), SC-560 (n = 4) (**d**,**g**,**j**,**m**), NS-398 (n = 4) (**e**,**h**,**k**,**n**), or SC-560 + NS-398 (n = 4) (**f**,**i**,**l**,**o**); n, number of bladder preparations. Each purine is expressed as a percentage of the total amount of purines detected in ILS at each time point of enzymatic reaction. AUC for time courses of eATP, eADP, eAMP, and eADO (**p**–**r**). Asterisks denote significant differences from vehicle control. * *p* < 0.05, ** *p* < 0.01, *** *p* < 0.001, **** *p* < 0.0001.

Next, we aimed to determine whether the inhibition of PGs production in the bladder mucosa would affect the degradation of eATP by s-NTDs in the ILS. Treatment with the COX-1 inhibitor SC-560 (COX-1 IC_50_ = 0.009 µM; COX-2 IC_50_ = 6.3 µM) [37]) alone led to increased eAMP and decreased eADO, while no changes in the eATP decrease or eADP increase were revealed (Figure 7b,d,g,j,m,p). Similarly, increased eAMP, decreased eADO, and unchanged eATP and eADP were observed in the ILSs of bladders treated with the selective COX-2 inhibitor NS-398 (COX-1 IC_50_ > 100 µM, COX-2 IC_50_ = 3.8 µM) [38]) (Figure 7b,e,h,k,n,q). However, in the ILSs of bladder preparations treated with the combination of SC-560 and NS-398, no changes in the time course of eATP decrease, and eADP, eAMP, and eADO increases were observed (Figure 7c,f,i,l,o). Only the eAMP AUC in the ILSs of bladders treated with SC-560 + NS-398 was greater than the eAMP AUC in vehicle controls (Figure 7r). Taken together, these results suggest that PGs produced in the bladder mucosa are indeed involved in the regulation of s-NTD release in the bladder lumen during filling.

## 3. Discussion

The present study provides the first evidence for PG-mediated modulation of the ATP hydrolysis by s-NTDs released in the bladder lumen. We found that (1) stimulation of DP1 and DP2 prostanoid receptors with either endogenous or exogenous PGD_2_ inhibits a step of the sequential hydrolysis of ATP to adenosine by s-NTDs released in the lumen during bladder filling, (2) exogenous PGE_2_ and PGI_2_, through activation of EP2 and IP prostanoid receptors, respectively, cause similar, although less prominent, effects on AMP formation from ATP as PGD_2_, and (3) endogenous PGs produced in the bladder mucosa by either COX-1 or COX-2 inhibit steps of the intravesical degradation of ATP by s-NTDs. The most prominent consequence of the PG action on the ATP degradation in the bladder lumen is the accumulation of AMP and delayed production of ADO due to an inhibited release of AMP-degrading soluble enzymes.

Upon distention, the bladder mucosa (urothelium and LP) produces an array of chemical mediators, including ATP and PGs, that regulate bladder excitability by affecting the functions of the urothelial, neural, interstitial, and detrusor smooth muscle cells within the bladder wall. ATP and PGs are released into the bladder lumen either constitutively or in response to distention, particularly during inflammation, acidosis, bladder overactivity, or mechanical trauma [27,39,40,41]. The majority of urinary ATP appears to originate in the bladder, whereas PGs (e.g., PGE_2_) may also be extracted from the plasma into the urine in the kidneys [42]. Regardless of their exact cellular origin, ATP and PGs can be simultaneously present in the bladder lumen and influence each other’s availability in the urine and within the bladder wall, ultimately affecting bladder excitability. As pointed out in the Introduction, intravesical ATP may be important for the accommodation of lumen expansion and urine storage during bladder filling by regulating umbrella cell membrane dynamics [16,43]. Intravesical instillation of ATP increases bladder activity [8,17], suggesting that the ATP that is present in the bladder lumen, through “transmural signaling”, could either activate purinergic receptors on cells deep in the bladder wall or induce the release of other mediators, ultimately initiating voiding [5,43]. Therefore, factors that modify the urinary levels of ATP could have a major impact on the overall bladder excitability. At present, it is unknown whether PGs alter the urinary levels of ATP.

ATP metabolism is a powerful and ubiquitous means for regulation of ATP availability at cellular and receptor targets in virtually all tissues in the body [44], including the bladder wall [3,4,45,46]. The ATP hydrolysis in the bladder LP and lumen is performed by both cell membrane-bound and soluble (releasable) NTDs [3,4]. In the bladder LP, s-NTDs are released spontaneously and more during bladder filling, suggesting that stretch induces s-NTDs release from the bladder mucosa. The release of s-NTDs in the LP is regulated by a number of membrane proteins, including stretch-activated channels and receptors that are targeted by urothelium-derived mediators [3,24,25,47]. As demonstrated previously [4] and in the present study, highly efficient s-NTDs are also released in the bladder lumen. It is important to acknowledge significant differences in the activities, temporal patterns of purine hydrolysis, and relative distribution of individual s-NTDs in the LP and the bladder lumen [3,4]. These differences suggest that the regulation of s-NTD release may vary on the opposite sides of the urothelium. For example, intravesical s-NTDs cause a fast disappearance of ATP, a transient appearance of ADP, and a gradual increase in AMP and ADO, whereas s-NTDs in the LP cause a gradual decrease in ATP and a gradual increase in ADP, AMP, and ADO. Differences in ATP hydrolysis might, at least in part, be responsible for the asymmetric distribution of ATP, ADP, AMP, and ADO in the LP and bladder lumen [48]. We recently demonstrated that the release of s-NTDs in the LP is differentially regulated by endogenous and exogenous PGs [29]. The present study aimed at determining whether PGs also regulate the s-NTDs release (and the consequent degradation of ATP) in the bladder lumen. This is a particularly important question, as both intravesical PGs and ATP appear to be increased in pathophysiological conditions such as bladder inflammation, overactive bladder syndrome, and bladder pain syndrome [5,30].

As in previous studies, we assessed the hydrolysis of ATP by adding a known amount/concentration of the highly-fluorescent analog of ATP, namely 1,*N*^6^-etheno-ATP/eATP, to the ILS that was removed from the bladder at the end of filling. We then evaluated the decrease in the eATP substrate and the increase in its products, eADP, eAMP, and eADO, at ten time points for the duration of one hour. It is validated that NTDs metabolize similarly etheno-derivatized and non-derivatized nucleotides [49,50]. However, the use of eATP provides 1,000,000-fold greater sensitivity and data reproducibility than the use of native ATP [51]. Importantly, the method ensures strict evaluation of eATP hydrolysis while endogenous purines that might be present in tissue samples remain below the detection limit. Moreover, the use of eATP as a substrate eliminates confusion by deamination because the etheno-bridge in 1,*N*^6^-etheno-derivatized purines blocks the N^6^ nitrogen, and thus, deaminases would not process ATP, ADP, AMP, or ADO to their inosine metabolites [49]. This is also supported by the observations in the present study that the concentrations of total e-purines remained constant for the duration of enzymatic reactions. Finally, assessing the time course of enzymatic reactions had clear advantages over assessing the eATP change at a single time point. This was particularly well-illustrated by the differences between the time courses of eAMP and eADO formation in the control ILS and the ILSs of treated bladders that would not be possible to observe by a single snapshot of eATP and e-product presence in ILS. To eliminate the potential impact of sources of s-NTDs in detrusor, upper urinary tract epithelium, or the kidneys, we carried out the study in a decentralized (ex vivo) murine bladder model with intact bladder mucosa and removed detrusor muscle [48]. This model has been used in many studies of spontaneous and distention-induced s-NTDs release in the bladder LP [3,24,25,29,47], including a study that determined the identities and basic characteristics of s-NTDs in the mouse bladder lumen [4]. Therefore, observations made in the present study can be directly compared with previous studies of s-NTDs release by the bladder mucosa.

Of the four assessed PGs, PGD_2_, PGE_2_, and PGI_2_ altered the degradation of eATP by s-NTDs released in the bladder lumen, whereas PGF_2α_ had no effects. PGD_2_, PGE_2_, and PGI_2_ increased the proportion of eAMP in the total purine pool, but the increase of eAMP was greater in the ILSs of bladders treated with PGD_2_ than in the ILSs of bladders treated with either PGE_2_ or PGI_2_. The formation of eADO was significantly reduced in the ILSs of bladders treated with PGD_2_, whereas the concentration of eADO in the ILSs of bladders treated with PGE_2_ or PGI_2_ was not significantly different from the vehicle controls. It is possible that the rather modest increase in eAMP in the presence of PGE_2_ or PGI_2_ could not be “translated” to detectable changes in the eADO concentrations. In the case of PGI_2_, for example, the IP receptor blockade did increase the eADO formation in the presence of PGI_2,_ suggesting that eADO tended to be decreased by PGI_2_ alone. A potential limitation of this study is the use of a single concentration of exogenous PGs (e.g., 10 µM), which may hinder possible differences between different PG concentrations. At the same time, however, data reported in the present study can be directly compared with observations made in other ex vivo studies of the bladder that use the same concentration of PGs (e.g., [29,52,53]). It should be pointed out that the actual concentrations of endogenous PGs at their sites of action (e.g., PG receptors) are largely unknown. Studies that report PGs as urinary biomarkers likely measure significantly degraded and diluted PGs. Compounds added to bath solutions commonly need to be applied at exceedingly high concentrations to reach the sites of action in effective concentrations. Therefore, comparing the effects of equimolar concentrations of different PGs, as in the present study, could provide valuable information about potential differences between PGs in a particular system.

In the present study, the increasing effects on the eAMP of exogenous PGE_2_ were mediated by the EP2 prostanoid receptor, whereas the effects of PGI_2_ were mediated by the IP prostanoid receptor. PGI_2_ is the dominant PG in the human bladder, but it is currently unclear if it has the same prominence in the mouse bladder. PGD_2_ had the most prominent effects on the release of s-NTDs that convert eAMP to eADO, causing an accumulation of eAMP and diminished formation of eADO. The effects of PGD_2_ were mediated by both DP1 and DP2 receptors. PGD_2_ is produced in immune cells and participates in the general development of inflammation, fibrosis, bronchospasm, and allergic reactions [54,55]. PGD_2_ has also been found to be released in the guinea pig bladder and appeared to inhibit detrusor muscle contractions caused by nerve stimulation [18]. However, its role in the bladder and specifically in the bladder mucosa is not understood.

Notably, there are considerable differences between the effects of exogenous PGs on s-NTDs release in the bladder lumen (reported here) and in the bladder LP that we demonstrated previously [29]. For example, in contrast to the effects in the ILS discussed above, in the LP, exogenous PGD_2_ and PGE_2_ increased the release of s-NTDs and accelerated the sequential degradation of eATP to eADO at multiple steps during the reactions. This is likely a self-guard mechanism attempting to mitigate excessive bladder excitability in response to high amounts of ATP and PGs released in disease states. These results reinforce the argument that measurements in the bladder lumen cannot be used as an indication of what occurs in the LP and other layers of the bladder wall. Further studies are warranted to elucidate the cellular and molecular mechanisms underlying the side-specific differences between s-NTDs release and PG action in the luminal and abluminal sides of the bladder mucosa.

The hydrolysis of AMP to ADO is performed by two main enzymes, namely 5-nucleotidase (NT5E/CD73) and alkaline phosphatase (ALPL)/tissue-nonspecific isozyme alkaline phosphatase (TNAP) [56,57]. Both enzymes are glycosylphosphatidylinositol (GPI)-anchored proteins that could be released after the cleavage of GPI by specific phospholipases [57,58]. We previously demonstrated that both NT5E and TNAP are released in ILS at the end of bladder filling [4]. Further studies are warranted to determine which AMPase—NT5E, ALPL/TNAP, or both—is inhibited by PGD_2_ and to a lesser degree by PGE_2_ and PGI_2_ in the bladder lumen. In any case, exogenous PGD_2_ should be expected to impede the formation of ADO in the bladder lumen. What would be the physiological/pathophysiological consequences of such events? It is generally accepted that ATP and ADO have the opposite effects on bladder excitability, so that ATP increases and ADO decreases detrusor muscle contractility [59]. Delayed formation of ADO from intravesical ATP, which is caused by PGs, may contribute to increased bladder excitability in cystitis and bladder pain syndrome. A recent study has suggested that prostacyclin (PGI_2_) signaling exacerbates cyclophosphamide-induced bladder inflammatory reactions [60]. The possibility that this might be due to PGI_2_-induced ATP/ADO imbalance in the bladder mucosa was not explored in this study.

Since the most prominent effects on eAMPase release were observed in response to exogenous PGD_2_, we explored whether stimulation of the DP1 and DP2 receptors by their endogenous ligand PGD_2_ would affect the release of s-NTDs in the ILS. Moreover, previous studies have reported the release of PGD_2_ [61] and the expression of DP1 and DP2 receptors [62] in the bladder mucosa. Inhibitions of either DP1 or DP2 receptors each caused the accumulation of eAMP and diminished the formation of eADO in ILSs containing eATP substrate. This similarity to the effects of the receptor agonist PGD_2_ was rather surprising. However, when the two DP receptors were simultaneously blocked, the alterations caused by the blockade of either DP1 or DP2 receptors were not observed. We concluded, therefore, that endogenous PGD_2_, just like exogenous PGD_2,_ can inhibit the release of s-AMPase(s), causing eAMP accumulation and eADO decrease via stimulation of either DP1 or DP2 receptors.

Overall, PG production appears to be low in healthy (e.g., uninflamed) tissues [63]. Even so, the PG-synthesizing enzyme COX-1 is constitutively expressed within the bladder wall, including the mucosa [30], and ongoing production of endogenous PGs contributes to bladder homeostasis [64]. Notably, the production of PGs seems to be far greater in the urothelium than in the smooth muscle [65,66]. PGs are released in response to stretch, nerve stimulation, or other mediators [27,67] and participate in the regulation of detrusor smooth muscle tone and release of other mediators in the urothelium [30], including ATP [68]. In the present study, selective inhibition of either COX-1 or COX-2 led to eAMP accumulation and diminished eADO formation. Based on these observations, one would assume that endogenous PGs that are produced by COX-1 or COX-2 should increase (not decrease) the intravesical release of s-AMPases. The combined inhibition of COX-1 and COX-2, however, abolished the effect of endogenous PGs on e-AMPase release and the consequent degradation of eAMP to eADO, suggesting that COX-1 and COX-2 simultaneously regulate the degradation of ATP to ADO by impeding the hydrolysis of AMP to ADO. COX-1 is generally described as a constitutive enzyme, whereas COX-2 is typically considered an inducible isoform of COX that is activated by pathophysiological stimuli, such as inflammation, hypoxia, or environmental toxins [69,70]. However, COX-2 is also constitutively expressed in certain cell types, including cells in the stomach [71], small intestine [72], the kidney, and the bladder [73], suggesting that COX-2 could be a factor in the basal production of PGs in the absence of pathophysiological stimuli. COX-2 can also be upregulated in response to urothelial cell stretch [74,75]. The majority of studies of mechanical stretch and COX-2 expression are focused on the role of COX-2 in nociception, inflammation, contractility, and proliferation caused by urinary tract obstruction [30]. Yet, the possibility that COX-2 may be activated during physiological bladder mucosa distention has not been eliminated. Our results suggest that both COX-1 and COX-2 are functionally expressed in the mouse bladder mucosa and complement each other in the urothelial production of PGs during bladder filling. These observations suggest functions for endogenous PGs not previously recognized to be present deep in the bladder wall or the bladder lumen.

**Figure 8:**
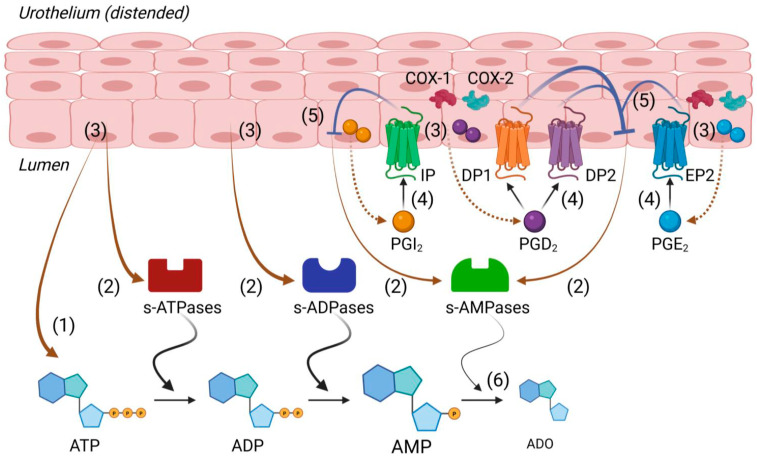
A model depicting regulation of s-NTDs release by endogenous and exogenous PGs. Distention of the bladder wall during bladder filling causes release of ATP (1), s-NTDs (e.g., s-ATPases, s-ADPases, s-AMPases) (2), and PGs (e.g., PGI_2_, PGD_2,_ and PGE_2_ that are produced locally by COX-1 and COX-2) (3) in the bladder lumen. S-NTDs sequentially break down ATP to ADP and AMP, and then, AMP is hydrolyzed to ADO. Released PGD_2_, PGE_2_, and PGI_2_, as well as exogenous PGs (or high concentrations of PGs released in the urine during inflammation), activate their G-protein coupled receptors (4) on membranes of umbrella cells and other urothelial cells. Activation of EP2 receptors by PGE_2_, of DP1 or DP2 receptors by PGD_2_, and of IP receptors by PGI_2_ triggers intracellular signaling mechanisms that inhibit the release of enzymes converting AMP to ADO (e.g., AMPases) (5). Diminished release of AMPases (e.g., NT5E and/or ALPL) leads to increased AMP and decreased ADO (6), likely causing prolonged excitation due to suppressed inhibition of bladder function. PG inhibition of s-NTDs/s-AMPases release in the bladder lumen is a novel mechanism of regulation of bladder function and can be targeted in disease states characterized by bladder overactivity or underactivity (Figure generated with BioRender).

In conclusion, the present study suggests that both endogenous and exogenous PGD_2_ (Figure 8) slow down the formation of ADO from extracellular ATP by inhibiting the release of soluble enzymes that break down AMP to ADO. It is possible that the delayed ADO production caused by endogenous PGD_2_ ensures the fine balance between the excitatory nucleotide ATP and the inhibitory nucleoside ADO that is necessary in the final stage of bladder filling. The pathophysiological implication of our observations could be that delayed ADO production by high concentrations of intraluminal PGs (e.g., mainly PGD_2_, and possibly PGE_2_, and PGI_2_), as expected in inflammation or bladder pain syndrome, could prolong the effects of excitatory extracellular purines in the bladder wall and increase bladder excitability. Further studies are warranted to determine the cellular and molecular mechanisms underlying the effects of PGs on s-NTDs release in the bladder lumen and the consequent degradation of ATP and AMP in the urine. Understanding how the release of s-NTDs is regulated may suggest novel ways to alter the effective concentrations of excitatory and inhibitory purines at receptor and cell targets in the bladder wall.

## 4. Materials and Methods

### 4.1. Animals

Male C57BL/6 mice (Jackson Laboratory, Bar Harbor, ME, USA; JAX stock #000664), 12–18 weeks of age, were reared in humidity- and temperature-controlled rooms under 12 h light–dark cycles and provided with water and standard chow ad libitum.

### 4.2. Ethical Approval

All studies outlined in this publication were acute studies. Mice were initially anesthetized by isoflurane inhalation, followed by cervical dislocation and then exsanguinated by decapitation. These procedures are in full accord with the recommendations of the Panel on Euthanasia of the American Veterinary Medical Association and the National Institutes of Health Guide for the Care and Use of Laboratory Animals. The techniques used to euthanize animals are humane and approved by the Institutional Animal Use and Care Committee at the University of Nevada, Reno (Protocol number: 20-09-1077-2).

### 4.3. Ex Vivo Mucosa-Only Bladder Preparation

Urinary bladders were harvested from euthanized animals and placed in ice-cold Krebs bicarbonate solutions (KBS) with the following composition (mM): 118.5 NaCl, 4.2 KCl, 1.2 MgCl_2_, 23.8 NaHCO_3_, 1.2 KH_2_PO_4_, 11.0 dextrose, and 1.8 CaCl_2_ (pH 7.4). In a dissecting dish filled with oxygenated KBS, fat, connective tissues, and bladder detrusor smooth muscle were removed by fine dissection, as described previously [3,4,48]. The bladder preparation was catheterized through the urethra with a PE-20 tubing and secured with double 6-0 silk and 6-0 nylon sutures. The bladder lumen was gently filled with KBS or a solution containing vehicle or drug using a 1 mL syringe to remove debris and estimate the approximate bladder capacity. Then, the empty bladder preparation was placed in a 3 mL water-jacketed chamber with a Sylgard-covered bottom, containing a working solution continuously supplied with a 95% O_2_/5% CO_2_ gas mixture and maintained at 37 °C.

### 4.4. Treatment of Bladder Preparations with Drugs

In experiments that were designed to evaluate how inhibition of COX-mediated production of endogenous PGs affects the release of s-NTDs in the bladder lumen and the consequent degradation of eATP in ILS collected from filled bladders, bladder preparations were incubated with the inhibitors throughout dissection, equilibration, and bladder filling. Similarly, bladder preparations were exposed to PG prostanoid receptor antagonists or COX inhibitors throughout dissection, equilibration, and bladder filling to ensure efficient blockade of PG receptors that mediate the effects of PGs. In studies that assessed the effects of exogenous PGE_2_, PGF_2α_, PGI_2_, and PGD_2_, dissection and equilibration were performed in either KBS or vehicle solution, and the drugs were applied in the bladder lumen only during bladder filling to avoid potential receptor desensitization. Table 1 summarizes the concentrations of the drugs used in these studies.

### 4.5. Collection of Intraluminal Solutions Containing s-NTDs

In the chamber, the bladder was equilibrated for 30 min, connected to an infusion syringe pump (Genie Touch, Kent Scientific, Torrington, CT, USA), and filled with working solution containing either vehicle or drug at a rate of 15 μL min^−1^ to its ~85 to 90% capacity. Such filling volumes were similar to the volumes that were necessary to generate pre-voiding intravesical pressure [48] and were considered pre-voiding volumes. Once the bladder was filled, the ILS was collected in a 600 µL Eppendorf tube for use in the enzymatic reaction.

### 4.6. Time-Course of eATP Hydrolysis in Intraluminal Solutions

The eATP substrate was prepared as described previously [3]. The time course of eATP degradation in ILS was evaluated as previously [4]. Briefly, concentrated eATP (50 µM) was added to an Eppendorf tube containing 200 µL ILS, resulting in a final eATP concentration of 2 µM. The tube with the reaction solution was kept at 37 °C for the duration of the enzymatic reactions using a water bath. At predetermined time points (10 s, 2′, 4′, 6′, 8′, 10′, 20′, 30′, 40′, and 60′), 20 µL aliquots of reaction solution were collected and immediately transferred into 300 µL HPLC polyethylene inserts containing 180 µL of ice-cold citric buffer (CB). The CB acidified the sample and terminated the enzymatic reaction at the particular time point, prevented potential spontaneous degradation of purines, and diluted the samples by a factor of 10. As a control for the reaction solution, 200 µL of working solution that had no prior contact with the bladder were combined with 2 µM eATP (called “beaker” sample) and processed in the same manner as the IL reaction solution aliquots. The inserts were placed in HPLC glass vials and stored at −20 °C until HPLC analysis with fluorescence detection was performed.

### 4.7. HPLC Analysis of eATP, eADP, eAMP, and eADO

The levels of the substrate eATP and its metabolites eADP, eAMP, and eADO in ILS collected at the end of bladder filling were measured using a reverse-phase gradient Agilent 1200 liquid chromatography with fluorescence detection (Agilent Technologies, Wilmington, DE, USA), as previously described [3,4,24,25,29,47]. To determine the amounts of eATP, eADP, eAMP, and eADO in each sample, the areas of chromatography signals/peaks were computed and plotted against standard curves generated with known concentrations of e-purines.

### 4.8. Drugs

The following drugs were used in these studies: from Sigma-Aldrich (St. Louis, MO, USA)—ATP, ADP, AMP, adenosine; from Bio-Techne Tocris (Minneapolis, MN, USA)—L-798,106, L-161,982, NS-398, PGE_2_, PGF_2α_, PGI_2_, PF04418948, SC51322, and SC560; from Cayman Chemicals (Ann Arbor, MI, USA)—PGD_2_, OC000459, and S-5751; and from MedChemExpress, (Monmouth Junction, NJ, USA)—RO1138452 and dimethyl sulfoxide (DMSO). All drugs were dissolved in DMSO and used at a final concentration of 0.2% DMSO.

### 4.9. Statistical Analyses of the Results

Calculations were performed using Excel (Microsoft Corporation, Redmond, WA, USA) and GraphPad Prism version 8.4.2 (GraphPad Software, San Diego, CA, USA). Data are presented as mean ± SD, and *p* values < 0.05 were considered statistically significant. Two-way analysis of variance (ANOVA) with Sidak’s or Tukey’s post hoc analysis was used for multiple comparisons. The area under the curve (AUC) for each time course was calculated using GraphPad Prism. AUCs from two or more groups were compared using an unpaired *t*-test or an ordinary one-way ANOVA, respectively.

## Figures and Tables

**Table 1:** Drugs and concentrations used.

Drug	Type	Concentration (µM)
PGE_2_	EP receptor agonist	10
PGD_2_	DP receptor agonist	10
PGI_2_ (Epoprostenol)	IP receptor agonist	10
PGF_2α_	FP receptor agonist	10
SC-560	COX-1 inhibitor	0.1
NS-398	COX-2 inhibitor	10
S-5751	DP1 receptor antagonist	1
OC51322	EP1 receptor antagonist	10
RO1138452	IP receptor antagonist	10
SC51322	EP1 receptor antagonist	1
PF04418948	EP2 receptor antagonist	1
L-798,106	EP3 receptor antagonist	0.25
L-161,982	EP4 receptor antagonist	1

## Data Availability

The raw data supporting the conclusion of this article will be made available by the corresponding author in the Dryad public data repository upon paper acceptance for publication (https://doi.org/10.5061/dryad.djh9w0w90).
